# The Prostate Health Index adds predictive value to multi-parametric MRI in detecting significant prostate cancers in a repeat biopsy population

**DOI:** 10.1038/srep35364

**Published:** 2016-10-17

**Authors:** V. J. Gnanapragasam, K. Burling, A. George, S. Stearn, A. Warren, T. Barrett, B. Koo, F. A. Gallagher, A. Doble, C. Kastner, R. A. Parker

**Affiliations:** 1 Academic Urology Group, University of Cambridge, Box 279 (S4) Cambridge Biomedical Campus Cambridge CB2 0QQ, UK; 2 CamPARI Clinic, Box 41, Clinic 4A, Cambridge University Hospitals Trust, Cambridge CB2 0QQ UK; 3 Department of Urology, Cambridge University Hospitals Trust, Cambridge CB2 0QQ UK; 4 Core Biochemical Assay Laboratory, Cambridge University Hospitals Trust, Cambridge CB2 0QQ UK; 5 Department of Pathology, Cambridge University Hospitals Trust,Cambridge CB2 0QQ UK; 6 Department of Radiology, University of Cambridge, Cambridge CB2 0QQ UK; 7 Department of Radiology, Cambridge University Hospitals Trust, Cambridge CB2 0QQ UK; 8 Edinburgh Clinical Trials Unit University of Edinburgh, Usher Institute of Population Health Sciences and Informatics, Edinburgh EH89AG UK.

## Abstract

Both multi-parametric MRI (mpMRI) and the Prostate Health Index (PHI) have shown promise in predicting a positive biopsy in men with suspected prostate cancer. Here we investigated the value of combining both tests in men requiring a repeat biopsy. PHI scores were measured in men undergoing re-biopsy with an mpMRI image-guided transperineal approach (n = 279, 94 with negative mpMRIs). The PHI was assessed for ability to add value to mpMRI in predicting all or only significant cancers (Gleason ≥7). In this study adding PHI to mpMRI improved overall and significant cancer prediction (AUC 0.71 and 0.75) compared to mpMRI + PSA alone (AUC 0.64 and 0.69 respectively). At a threshold of ≥35, PHI + mpMRI demonstrated a NPV of 0.97 for excluding significant tumours. In mpMRI negative men, the PHI again improved prediction of significant cancers; AUC 0.76 vs 0.63 (mpMRI + PSA). Using a PHI≥35, only 1/21 significant cancers was missed and 31/73 (42%) men potentially spared a re-biopsy (NPV of 0.97, sensitivity 0.95). Decision curve analysis demonstrated clinically relevant utility of the PHI across threshold probabilities of 5–30%. In summary, the PHI adds predictive performance to image-guided detection of clinically significant cancers and has particular value in determining re-biopsy need in men with a negative mpMRI.

The Prostate Health Index (PHI) was developed by incorporating the pro-2PSA, free PSA and total PSA into a mathematical algorithm^1^. Le *et al*. published one of the first papers evaluating the PHI in 2034 men and found that it significantly improved prediction of a positive biopsy compared to PSA in the range of 2.5–10 ng/ml^2^. Since then, many others have consistently shown similar results with different PSA ranges and across different populations^3^,^4^,^5^,^6^. Most recently, the PHI and 4K score were evaluated in the Swedish STHLM2 trial and both were shown to increase prediction of aggressive cancers and spare about a third of men an unnecessary biopsy^7^. The PHI is now approved by the US FDA and been adopted into the US NCCN guidelines^8^,^9^.

Despite these promising results, the PHI is not yet in widespread use and an unanswered question is how it fits with the increasing use of pre-biopsy imaging. Multi-parametric MRI (mpMRI) has emerged as an important tool for both identifying men who need a biopsy and where to target the needles^10^,^11^. This is particularly important in a repeat biopsy population where mpMRI guidance has shown superiority over other approaches in predicting a positive finding when a prior biopsy has been negative or thought to have under estimated the disease burden^12^. mpMRI however, cannot always reliably exclude cancers. Indeed, systematic reviews have shown a wide range of negative predictive values (NPV) for even significant cancer detection (63–98%)^13^,^14^. Work in our own unit has shown very similar findings in a repeat biopsy population with NPV of 66–89% depending on radiologist experience^15^,^16^. As a result, many men are likely to still undergo a repeat prostate biopsy despite a negative mpMRI. This is an unnecessary risk to patients and significant waste of resources. In this context a blood based assay (such as the PHI) to identify men who will most benefit from an mpMRI guided biopsy would be very useful.

In this study we explored the potential value of the PHI in the context of image guided repeat biopsies. Our specific aims were to assess the value of the PHI in predicting a positive mpMRI scan, test its incremental value to mpMRI in predicting cancers and its utility in deciding which men with a negative mpMRI should proceed with a re-biopsy.

## Materials and Methods

### Patients, imaging and biopsy data

Between 2013 and 2015, men due to undergo a repeat prostate biopsy by a transperineal approach were recruited into a prospective study (Ethics 03/018 - NRES Committee East of England, UK) in our centre with informed consent obtained from all participants. The experimental protocol for patient recruitment, sample handling and processing, use of imaging and data were approved by this body. All methods were therefore performed in accordance with the relevant guidelines and regulations.

All men had previously had at least 1 prostate biopsy and required re-biopsy because of ongoing suspicion of cancer or where cancer grade had thought to be underestimated. All had a prior mpMRI and proceeded to biopsy regardless of whether a target lesion was identified or not. For the purposes of this study men with any suspicion of extra capsular disease (on mpMRI), infection, prostatitis or previous prostate surgery/treatment were excluded. A total of 279 men were included in this analysis. Patients underwent mpMRI on a 1.5T MR450 or 3T Discovery MR750-HDx system (GE Healthcare, USA) with a multi-channel surface phased array coil, including standard anatomical and functional diffusion-weighted imaging using multiple b-values, as previously described^17^. Image acquisition and processing was performed in accordance with standard clinical mpMRI protocols in the Department of Radiology, Addenbrookes Hospital. T2W and DWI sequences were evaluted and scored using a Likert scale of cancer probability, based on the Prostate Imaging Reporting and Data (PI-RADS) descriptors developed by the European Society of Urogenital Radiology (ESUR)^18^. This study however pre-dated the PI-RADS 2 revision. All images were reviewed and discussed in a Multi-Disciplinary Team (MDT) meeting before biopsy. Prostate volumes were calculated from mpMRI images. The contours of mpMRI defined lesions (Likert 3–5) were drawn on the Biopsee^TM^ fusion platform (Medcom, Germany). No lesion or Likert 1–2 lesions were considered mpMRI negative for this study. 24 sectoral biopsies +2 from each target area using image fusion guidance (where applicable) were taken using a previously published standardised template (Ginsberg protocol)^19^. Men without mpMRI lesions (mpMRI negative) had 24 sectoral biopsies taken only. All procedures were done by 1 of 3 urologists with several years’ experience of transperineal biopsy. Biopsy samples were processed through a single pathology laboratory and reported by specialist uro-pathologists (using ISUP 2005) and re-reviewed in the (MDT) meeting before a final grade and diagnosis was assigned.

### PHI assay

The protocol for performing the PHI assay was performed according to the manufactures guidelines and recommendations (Beckman Coulter). Blood was taken prior to biopsies, and at least four weeks after any prostate manipulation. Samples were centrifuged and frozen at −80c within three hours. PHI assays were performed on the Beckman Coulter Access autoanalyser. Quality Assurance samples were analysed before and after each batch to ensure the validity of the results. All reagents, calibrators and QC’s were purchased from Beckman Coulter. All QC results were within Beckman Coulter’s target ranges.

### Statistical analysis

The PHI was evaluated in the primary cohort for predicting (i) any cancer, and (ii) any significant cancer in combination with mpMRI. For this study, and to be consistent with current guideline definitions of risk, we defined clinical significance as any Gleason sum ≥7 disease^8^,^20^. No PHI threshold was pre-determined before the study. Logistic regression models were used to generate receiver operative curves for the variables of mpMRI alone, mpMRI + PSA and mpMRI + PHI. Differences in area-under-the-curve (AUC) were calculated using Delong’s method in the “pROC” package in R software^21^,^22^. Diagnostic test statistics (sensitivity, specificity, positive predictive value [PPV], negative predictive value [NPV], number of unnecessary biopsies, and number with cancer missed) were derived to identify the optimum PHI threshold for cancer detection. This analysis sequence was then repeated in the cohort of men with a negative mpMRI. Decision curve analysis (DCA) were used to investigate the clinical value of the test strategies^23^. Finally we included a sensitivity analysis to exclude men who already had a cancer diagnosis before re-biopsy and retested our results. Analysis was performed using R software version 3.2.3 and SPSS version 21^22^,^24^.

## Results

### Cohort description and value of PHI in predicting a mpMRI lesion

The demographic details of the primary cohort are shown in Table 1. 279 men were included the majority of whom had 1–2 prior prostate biopsies. Most had no previous cancer diagnosis, although 65/279 (23%) underwent a repeat biopsy to exclude more extensive disease. Table 2 details the distribution of the PHI components stratified by mpMRI findings and pre-biopsy histology. At mpMRI 185/279 (66%) men had a Likert ≥3 lesion and 94 (34%) had no lesion or only Likert 1–2. We first tested if the PHI could predict if an mpMRI would be positive. PHI scores were generally higher in men with a mpMRI lesion (Table 2). However, using the PHI only marginally increased predictive value compared to PSA; Area under the curve (AUC) for PHI of 0.60 [0.53–0.67] for any Likert score (vs 0.47 [0.40–0.54] for PSA) and 0.59 [0.52–0.66] for a Likert 4/5 lesion (vs. 0.51 [0.44–0.58] for PSA). These results suggest that the PHI is unlikely to be useful as a triaging test in deciding if an mpMRI will be positive.

### Value of the PHI in addition to mpMRI in predicting biopsy outcome

**Figure 1:**
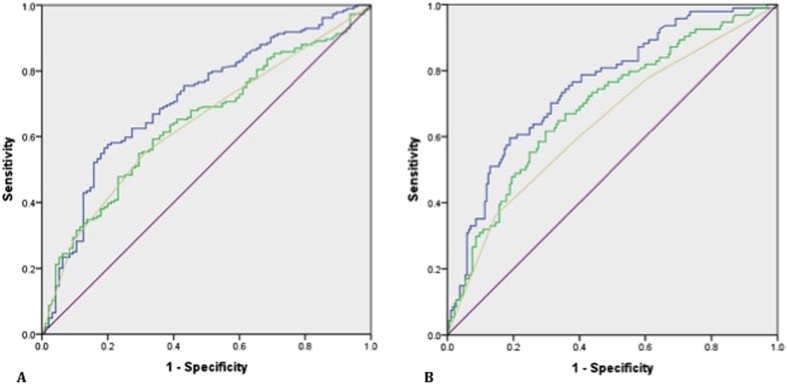
Area under the curve (AUC) comparison of performance of the PHI in combination with mpMRI in identifying men who will be positive on a repeat biopsy for (**A**). Any cancer and (**B**). Significant (Gleason sum ≥7) cancers. (n = 279). Purple –reference, Yellow – mpMRI only, Green - mpMRI + PSA, Blue - mpMRI + PHI.

**Figure 2:**
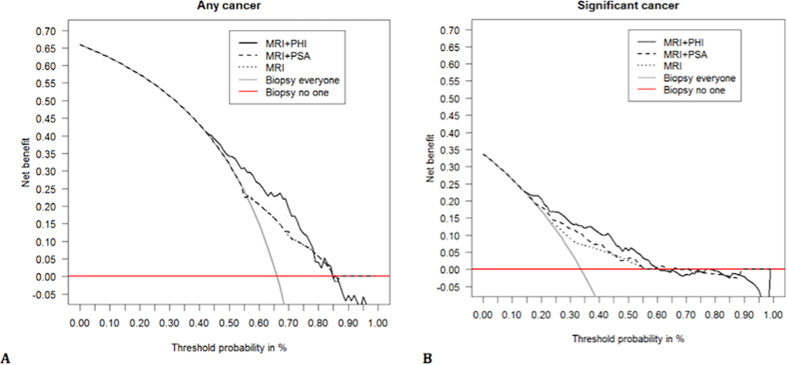
Decision curve analysis comparing the added value of the PHI test in addition to mpMRI in identifying men who will be positive on a repeat biopsy for (**A**). Any cancer and (**B**). Significant (Gleason sum ≥ 7) cancers. (n = 279).

Following repeat biopsies, 95 (34%) men had a benign outcome and 184 (66%) had cancers detected of whom 94 (34%) were Gleason ≥7. The PHI alone was a significant predictor for any cancer (OR 1.018, 95% CI 1.004–1.032, p = 0.01) and significant cancers (OR 1.025, 95% CI 1.013–1.038, p < 0.0001) after adjusting for mpMRI in logistic regression. Adding PHI to mpMRI also improved predictive performance for overall cancer detection; AUC 0.71 [0.61–0.76] vs. 0.64 (mpMRI) (Fig. 1A). In comparison, adding PSA alone to mpMRI did not appreciably alter predictive performance (AUC 0.64). We next focused on prediction of only significant cancers. Adding PHI to mpMRI, again showed a much greater improvement in predictive performance (AUC 0.75, vs. 0.64) (Fig. 1B). In contrast, adding PSA to mpMRI only marginally improve positive biopsy prediction compared to mpMRI alone (AUC 0.69 vs. 0.64) (Fig. 1B). These results were very similar and conclusions unchanged after excluding patients with previous cancer. To contextualise the improvement in predictive value, we plotted decision curves. For overall cancer detection, decision curve analysis (DCA) showed that the PHI only added value to mpMRI in decision making for threshold probabilities of 45–85% which would be unlikely to be of clinical use (Fig. 2A). DCA for significant cancers, however, showed that the PHI did add value with a net benefit across threshold probabilities as low as 15% which would be clinically useful (Fig. 2B). These threshold probability ranges were identical in sensitivity analysis excluding patients with previous cancer. To determine an optimal PHI level we next tested different cut-off values in detecting or missing cancers. For overall cancer prediction no specific PHI cut-off showed promise (Supplementary Table 1). For significant cancers, however, a PHI ≥35 would have detected all but one tumour with a NPV of 0.97 and sensitivity of 0.99 (Table 3). After excluding patients with previous cancer, the results were again very similar.

### Use of the PHI score in determining biopsy need in men with a negative mpMRI

**Figure 3:**
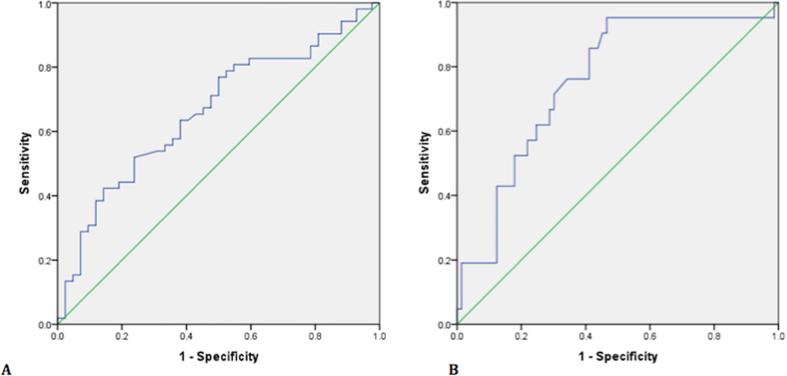
Area under the curve (AUC) comparison of performance of the PHI in men with a negative mpMRI in identifying men who will be positive on a repeat biopsy for (**A**). Any cancer AUC 0.66 (95% CI 0.55 to 0.77) and B. Significant (Gleason sum ≥7) cancers 0.76 (95% CI 0.64 to 0.87). (n = 94). Green – reference, Blue – PHI.

**Figure 4:**
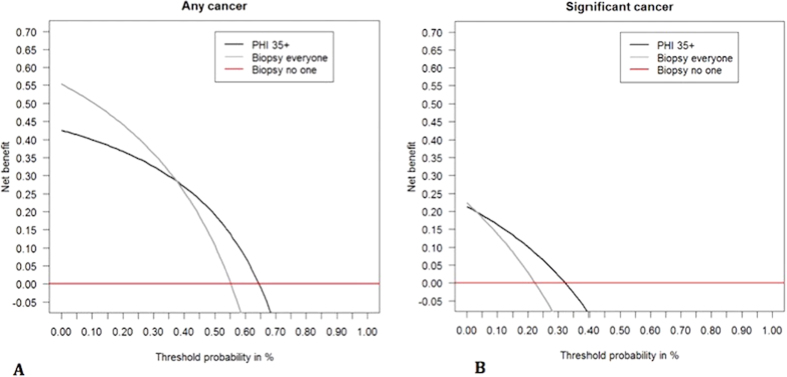
Decision curve analysis testing the performance of the PHI threshold of ≥35 in men with a negative mpMRI (n = 94) in identifying men who will be positive on a repeat biopsy for (**A**). Any cancer and (**B**). Significant (Gleason sum ≥7) cancer.

Amongst the 94 negative mpMRI, 52 cancers were diagnosed including 21 Gleason ≥7. In this group, the PHI had an AUC of 0.66 for overall cancer detection (95% CI 0.55–0.77), but was particularly useful in predicting significant cancers (AUC 0.76, 95% CI 0.64 – 0.87) and outperformed PSA and PSA density (Fig. 3A,B and Supplementary Table 2). After excluding patients with a prior cancer diagnosis, the PHI AUC increased to 0.79 (95% CI 0.65 to 0.93) for predicting significant cancers. A PHI threshold of ≥35 showed the best discriminatory value missing only 1/21 Gleason ≥7 tumour and potentially sparing 42% of the remaining cohort from a biopsy episode (31/73) (Table 4). Of note the missed cancer only involved 10% of a single core out of 24 samples and therefore represented minimal disease. Finally, to assess the clinical improvement in predictive value, we repeated a DCA. This again did not show much benefit for overall cancer detection, but did so for predicting significant cancers across threshold probabilities from 5–30% (Fig. 4A,B). This is a clinically very relevant uncertainty range when deciding if a biopsy is necessary with a negative mpMRI. A sensitivity analysis excluding patients with previous cancer gave very similar results in the DCA.

## Discussion

Research into new diagnostics in prostate cancer have focussed on two main areas (i) mpMRI for image guided biopsies and (ii) biomarkers to better predict the presence of cancer. To date, there has been a paucity of research that has combined these two aspects in well-structured cohort studies, particularly, in the re-biopsy setting^25^,^26^. As a result, there is significant uncertainty as to how to maximise the benefits of both in clinical practice. Head-to-head comparisons have not surprisingly shown that biomarkers alone fare worse than imaging in predicting cancers^25^,^27^,^28^. Only one study has tested both imaging and the PHI in the re-biopsy context. Porpiglia *et al*. compared performance of the PHI and PCA3 with mpMRI in a repeat biopsy population^29^. In this study, neither PHI nor PCA3 added to the value of mpMRI in predicting cancers. Crucially however, the biopsies were done blinded to the mpMRI findings and were performed transrectally. We have previously shown that image guided and transperineal biopsies are significantly better in detecting cancers in the re-biopsy context and are a better reference test^12^. Indeed, a recent UK Health Technology Assessment of the PHI identified the lack of good biopsy reference standards as a weakness of previous comparative studies^25^. Other identified uncertainties from the paper included the value of the PHI in (i) predicting an mpMRI lesion, (ii) predicting significant cancers and (iii) the optimal sequence for using the PHI and mpMRI in practice^25^. Our current study addresses all these uncertainties in its design and is the first to unify a comprehensive mpMRI fusion guided transperineal biopsy protocol with evaluation of the PHI as a serum diagnostic biomarker.

mpMRI is known to have a high specificity and sensitivity for detecting clinically significant cancers^30^,^31^. However, it is an expensive and resource intensive investigation and unlikely to be suitable as a screening tool. An important question therefore is if the PHI (or other biomarkers) might be used as a triaging test to predict a positive mpMRI. In this study, although PHI scores were generally higher when an mpMRI was positive, it was not reliable in predicting a lesion. One caveat is that these findings are in a re-biopsy cohort and it may not equally apply in a biopsy naïve population. We next asked if the PHI could improve mpMRI predictive performance in the context of transperineal image guided biopsies. In terms of overall detection, our findings agree with those of Porpiglia *et al*. in that the PHI added little additional clinical value^29^. However, the PHI did improve predictive performance for significant cancers, particularly using a cut-off ≥35 and this is likely to be clinically relevant. This level was not predefined for this study, instead it was derived by testing different thresholds to identify the optimal value. Indeed, no previous study has established the optimal PHI for a repeat biopsy population, although one study (using a non-image guided approach) identified a correlation with significant cancer detection and a PHI ≥55^32^. A PHI ≥35 is higher than that reported in first biopsy studies but is actually the threshold recommended by the NCCN guidelines^8^.

Re-biopsies in men with a negative mpMRI is controversial and there is currently no consensus or agreed approach. Many urologists might not advocate a re-biopsy especially if the PSA density is low^33^. mpMRI reporting however can be highly variable and the rate of significant cancers despite a negative mpMRI is not inconsiderable^11^,^13^,^16^,^30^. In our cohort (with experienced uro-radiologists) significant cancers were found in 22% of mpMRI negative men. Amongst these men, a PHI ≥35 identified all but one very low volume and minimal Gleason ≥7 cancer. Decision curve analysis further suggested that the PHI was clinically useful across a relevant threshold of uncertainty. In practice nearly half of men with a negative mpMRI men in this study might have potentially been safely spared a biopsy.

Our study has some inherent limitations. It is single centre and includes relatively modest numbers. Our study however benefit from a well-characterised cohort, use of image-guided transperineal biopsies and being undertaken by an experienced multi-disciplinary diagnostic team^12^,^15^,^16^,^17^. The findings may not be similar in less experienced units or with other biopsy approaches and further validation in a multi-institutional setting is needed. Our study was also based in a repeat biopsy population and needs re-testing in a first biopsy population, which we are currently undertaking. Our mpMRI didn’t include dynamic contrast enhancement, however we note that its added value to lesion scoring is currently doubtful^34^. Our study also did not use the updated PIRADS version 2 as our cohort predated this publication. Our criteria for clinical significance was based on any detection of Gleason 7 disease and did not account for the number of cores positive or percentage cores positive. Our rationale was based on the fact that current risk stratification criteria (UK NICE, AUA, NCCN) do not yet include these latter elements and our sample size was not large enough to further sub-divide the cohorts. Finally, we did not employ study-specific repeat review of all imaging and histology. However, all our results are re-reviewed in a multidisciplinary meeting setting and this is more representative of real world clinical pathways.

In summary we show for the first time that combing the serum biomarker PHI to imaging (mpMRI) improves the predictive ability for detection significant prostate cancer but not for overall cancer detection. The PHI may have particular value in identifying men who do need a biopsy when the mpMRI is negative. In this regard, the PHI might help reduce the number of unnecessary biopsies especially where there is either limited or evolving experience in reading mpMRIs. These findings are preliminary and do need further validation in future larger studies. However they do suggest first evidence of a complementary role for the PHI assay with mpMRI in image-guided re-biopsies. Our next studies will explore whether this relationship holds true in a first biopsy population. This will be important in the context of on-going studies which are evaluating both the PHI and mpMRI as first line screening tests.

## Additional Information

**How to cite this article**: Gnanapragasam, V. J. *et al*. The Prostate Health Index adds predictive value to multi-parametric MRI in detecting significant prostate cancers in a repeat biopsy population. *Sci. Rep.* **6**, 35364; doi: 10.1038/srep35364 (2016).

## Supplementary Material

Supplementary Information

## Figures and Tables

**Table 1:** Descriptive characteristics of the primary study cohort prior to template biopsies. mpMRI (multi-parametric MRI).

Patient data (n = 279)	Median (range)
Age (years)	66 (45–80)
Prostate volume (mls)	52 (11–230)
mpMRI lesion	
No target/Likert 1–2	94 (34%)
Likert 3	53 (19%)
Likert 4	71 (25%)
Likert 5	61 (22%)
Prior cancer on biopsy	
No	214 (77%)
Yes – Gleason 6	60 (22%)
Yes – Gleason 7*	5 (2%)

^*^Gleason 7 (3 + 4) cases before repeat biopsy involved ≤2 cores with <10% tumour involvement.

**Table 2:** Distribution of median and range of PHI derivative components, derived PHI score and PSA density in the whole cohort (n = 279) stratified by pre-repeat biopsy mpMRI findings and histology.

Variable median (range)	mpMRI			Pre- biopsy histology		
	No lesion/Likert 1–2	Likert 3–5	Likert 4–5	No cancer	Gleason ≥6	Gleason ≥7
	(n = 94)	(n = 185)	(n = 132)	(n = 214)	(n = 60)	(n = 5)
**tPSA**	9.37 (2.10–30.4)	9.14 (0.30–40.5)	9.31 (0.30–40.5)	9.60 (0.30–40.5)	9.08 (0.95–22.2)	7.69 (4.20–10.6)
**Free PSA**	1.48 (0.30–9.30)	1.20 (0.10–5.62)	1.20 (0.10–5.62)	1.36 (0.10–9.30)	1.30 (0.23–5.20)	0.60 (0.30–1.07)
**p2PSA**	18.6 (4.80–143.2)	18.3 (2.77–96.3)	17.8 (2.77–96.3)	18.6 (4.77–143.2)	18.5 (2.77–58.7)	10.4 (9.71–15.7)
**PHI**	38.2 (13.1–214.1)	44.4 (11.7–219.6)	45.3 (11.7–219.6)	40.5 (13.1–219.6)	44.7 (11.7–135.5)	38.1 (33.3–119.4)
**PSA density**	0.19 (0.05–0.64) (n = 71)	0.28 (0.02–1.57) (n = 148)	0.29 (0.02–1.57) (n = 109)	0.24 (0.02–1.57) (n = 169)	0.25 (0.06–0.76) (n = 47)	0.24 (0.08–0.34) (n = 3)

Data on PSA density was not available in some of the cohort.

**Table 3:** Diagnostic test statistics modeling each of the different strategies incorporating various PHI thresholds and detection rates for significant cancers (Gleason sum ≥7).

Decision to biopsy	Sensitivity (95% CI)	Specificity (95% CI)	PPV (95% CI)	NPV (95% CI)	No. of non-significant biopsies	No. of missed Gl ≥ 7
mpMRI positive	0.78 (0.68–0.86)	0.39 (0.32–0.47)	0.39 (0.32–0.47)	0.78 (0.68–0.86)	112	21
mpMRI positive or PHI ≥ 25	0.99 (0.94–1)	0.04 (0.02–0.08)	0.34 (0.29–0.40)	0.89 (0.52–1)	177	1
mpMRI positive or PHI ≥ 30	0.99 (0.94–1)	0.11 (0.067–0.16)	0.36 (0.30–0.42)	0.95 (0.76–1)	165	1
mpMRI positive or PHI ≥ 35	0.99 (0.94–1)	0.17 (0.12–0.23)	0.38 (0.32–0.44)	0.97 (0.84–1)	154	1
mpMRI positive or PHI ≥ 40	0.95 (0.88–0.98)	0.25 (0.19–0.32)	0.39 (0.33–0.46)	0.90 (0.79–0.97)	138	5

Non-significant biopsies combine negative biopsies and those detecting Gleason sum 6 cancers only. mpMRI positive refers to lesions of Likert 3 or greater.

**Table 4:** Diagnostic test statistics modeling each of the different strategies incorporating various PHI thresholds and detection rates for significant cancers (Gleason sum ≥7) in men with a negative mpMRI (n = 94).

Decision to biopsy	Sensitivity (95% CI)	Specificity (95% CI)	PPV (95% CI)	NPV (95% CI)	No. of non-significant biopsies	No. of missed Gl ≥ 7
PHI ≥ 25	0.95 (0.76–1)	0.11 (0.049–0.2)	0.24 (0.15–0.34)	0.89 (0.52–1)	65	1
PHI ≥ 30	0.95 (0.76–1)	0.27 (0.18–0.39)	0.27 (0.18–0.39)	0.95 (0.76–1)	53	1
PHI ≥ 35*	0.95 (0.76–1)	0.42 (0.31–0.55)	0.32 (0.21–0.45)	0.97 (0.84–1)	42	1#
PHI ≥ 40	0.76 (0.53–0.92)	0.64 (0.52–0.75)	0.38 (0.24–0.54)	0.90 (0.79–0.97)	26	5

^*^The model of proceeding to biopsy only if the PHI was ≥35 gave the optimum performance. Non-significant biopsies combine negative biopsies and those detecting Gleason sum 6 cancers only.

^#^Missed tumour was Gleason 3 + 4 in only 1/24 cores, <10% core involvement.

## References

[b1] Catalona W. J. . Serum pro-prostate specific antigen preferentially detects aggressive prostate cancers in men with 2 to 4 ng/ml prostate specific antigen. J Urol. 171, 2239–2244 (2004). 15126794 10.1097/01.ju.0000127737.94221.3e

[b2] Le B. V. . [−2]Proenzyme prostate specific antigen is more accurate than total and free prostate specific antigen in differentiating prostate cancer from benign disease in a prospective prostate cancer screening study. J Urol. 183, 1355–1359 (2010). 20171670 10.1016/j.juro.2009.12.056 PMC3537165

[b3] Catalona W. J. . A multicenter study of [−2]pro-prostate specific antigen combined with prostate specific antigen and free prostate specific antigen for prostate cancer detection in the 2.0 to 10.0 ng/ml prostate specific antigen range. J Urol. 185, 1650–1655 (2011). 21419439 10.1016/j.juro.2010.12.032 PMC3140702

[b4] Stephan C. . Multicenter evaluation of [−2]proprostate-specific antigen and the prostate health index for detecting prostate cancer. Clin Chem. 2013 59, 306–314 (2013). 10.1373/clinchem.2012.195784 23213080

[b5] de la Calle C. . Multicenter Evaluation of the Prostate Health Index to Detect Aggressive Prostate Cancer in Biopsy Naïve Men. J Urol. 194, 65–72 (2015). 25636659 10.1016/j.juro.2015.01.091 PMC4696043

[b6] Wang W. . Diagnostic ability of %p2PSA and prostate health index for aggressive prostate cancer: a meta-analysis. Sci Rep. 4, 5012, 10.1038/srep0501 (2014). 24852453 PMC5381367

[b7] Nordström T. . Comparison Between the Four-kallikrein Panel and Prostate Health Index for Predicting Prostate Cancer. Eur Urol. 68, 139–146 (2015). 25151013 10.1016/j.eururo.2014.08.010 PMC4503229

[b8] National Comprehensive Cancer Network Clinical Practice Guidelines in Oncology. Prostate Cancer Early Detection Version 2014. http://www.nccn.org/professionals/physician_gls/pdf/prostate_detection.pdf. Accessed April 10^th^, (2016).

[b9] Lepor A., Catalona W. J. & Loeb S. The Prostate Health Index: Its Utility in Prostate Cancer Detection. Urol Clin North Am. 43, 1–6 (2016). 26614024 10.1016/j.ucl.2015.08.001 PMC4663012

[b10] Schoots I. G. . Magnetic resonance imaging-targeted biopsy may enhance the diagnostic accuracy of significant prostate cancer detection compared to standard transrectal ultrasound-guided biopsy: a systematic review and meta-analysis. Eur Urol. 68, 438–450 (2015). 25480312 10.1016/j.eururo.2014.11.037

[b11] de Rooij M., Hamoen E. H., Fütterer J. J., Barentsz J. O. & Rovers M. M. Accuracy of multiparametric MRI for prostate cancer detection: a meta-analysis. AJR Am J Roentgenol. 202, 343–351 (2014). 24450675 10.2214/AJR.13.11046

[b12] Nelson A. W. . Repeat prostate biopsy strategies after initial negative biopsy: meta-regression comparing cancer detection of transperineal, transrectal saturation and MRI guided biopsy. PLoS One. 8, e57480 10.1371/journal.pone.0057480 (2013). 23460864 PMC3583836

[b13] Fütterer J. J. . Can Clinically Significant Prostate Cancer Be Detected with Multiparametric Magnetic Resonance Imaging? A Systematic Review of the Literature. Eur Urol. 68, 1045–1053 (2015). 25656808 10.1016/j.eururo.2015.01.013

[b14] Zhang Z. X. . The value of magnetic resonance imaging in the detection of prostate cancer in patients with previous negative biopsies and elevated prostate-specific antigen levels: a meta-analysis. Acad Radiol. 21, 578–589 (2014). 24703470 10.1016/j.acra.2014.01.004

[b15] Gaziev G. . Defining the learning curve for multiparametric magnetic resonance imaging (MRI) of the prostate using MRI-transrectal ultrasonography (TRUS) fusion-guided transperineal prostate biopsies as a validation tool. BJU Int. 117, 80–86 (2016). 25099182 10.1111/bju.12892

[b16] Serrao E. M. . Investigating the ability of multiparametric MRI to exclude significant prostate cancer prior to transperineal biopsy. Can Urol Assoc J. 9, 11–12 (2015). 26788234 10.5489/cuaj.2895 PMC4707904

[b17] Lawrence E. M. . Prostate cancer: performance characteristics of combined TW and DW-MRI scoring in the setting of template transperineal re-biopsy using MR-TRUS fusion. Eur Radiol. 7, 1497–1505 (2014). 10.1007/s00330-014-3159-0 24744197

[b18] Barentsz J. O. . European Society of Urogenital Radiology. ESUR prostate MR guidelines 2012. Eur Radiol. 22, 746–757 (2012). 22322308 10.1007/s00330-011-2377-y PMC3297750

[b19] Kuru T. H. . Definitions of terms, processes and a minimum dataset for transperineal prostate biopsies: a standardization approach of the Ginsburg Study Group for Enhanced Prostate Diagnostics. BJU Int. 112, 568–577 (2013). 23773772 10.1111/bju.12132

[b20] NICE. Prostate cancer: diagnosis and treatment. NICE guidelines [CG175]. (2014).

[b21] Xavier Robin . pROC: an open-source package for R and S+ to analyze and compare ROC curves. BMC Bioinformatics. 12, 77 10.1186/1471-2105-12-77 (2011). 21414208 PMC3068975

[b22] R Core Team R: A language and environment for statistical computing. R Foundation for Statistical Computing, Vienna, Austria. URL https://www.R-project.org/ (2015).

[b23] Vickers A. J. Decision analysis for the evaluation of diagnostic tests, prediction models and molecular markers. Am Stat. 62, 314–320 (2008). 19132141 10.1198/000313008X370302 PMC2614687

[b24] Corp I. B. M.. Released IBM SPSS Statistics for Windows, Version 21.0. Armonk, NY: IBM Corp. (2012).

[b25] Nicholson A. . The clinical effectiveness and cost-effectiveness of the PROGENSA^®^ prostate cancer antigen 3 assay and the Prostate Health Index in the diagnosis of prostate cancer: a systematic review and economic evaluation. Health Technol Assess. 19, 1–191 (2015). 10.3310/hta19870 PMC4780983 26507078

[b26] Scattoni V. . Head-to-head comparison of prostate health index and urinary PCA3 for predicting cancer at initial or repeat biopsy. J Urol. 2013 190, 496–501 (2013). 23466239 10.1016/j.juro.2013.02.3184

[b27] Lazzeri M. . Serum isoform [-2]proPSA derivatives significantly improve prediction of prostate cancer at initial biopsy in a total PSA range of 2-10 ng/ml: a multicentric European study. Eur Urol. 63, 986–994 (2013). 23375961 10.1016/j.eururo.2013.01.011

[b28] Sciarra A. . Multiparametric magnetic resonance imaging of the prostate can improve the predictive value of the urinary prostate cancer antigen 3 test in patients with elevated prostate-specific antigen levels and a previous negative biopsy. BJU Int. 110, 1661–1665 (2012). 22564540 10.1111/j.1464-410X.2012.11146.x

[b29] Porpiglia F. . The roles of multiparametric magnetic resonance imaging, PCA3 and prostate health index-which is the best predictor of prostate cancer after a negative biopsy? J Urol. 192, 60–66 (2014). 24518780 10.1016/j.juro.2014.01.030

[b30] Vos E. K. . Multiparametric Magnetic Resonance Imaging for Discriminating Low-Grade From High-Grade Prostate Cancer. Invest Radiol. 50, 490–497 (2015). 25867656 10.1097/RLI.0000000000000157

[b31] Hamoen E. H., de Rooij M., Witjes J. A., Barentsz J. O. & Rovers M. M. Use of the Prostate Imaging Reporting and Data System (PI-RADS) for Prostate Cancer Detection with Multiparametric Magnetic Resonance Imaging: A Diagnostic Meta-analysis. Eur Urol. 67, 1112–1121 (2015). 25466942 10.1016/j.eururo.2014.10.033

[b32] Boegemann M. . The percentage of prostate-specific antigen (PSA) isoform [-2]proPSA and the Prostate Health Index improve the diagnostic accuracy for clinically relevant prostate cancer at initial and repeat biopsy compared with total PSA and percentage free PSA in men aged ≤65 years. BJU Int. 117, 72–79 (2016). 25818705 10.1111/bju.13139

[b33] Washino S. . Combination of PI-RADS score and PSA density predicts biopsy outcome in biopsy naïve patients. BJU Int. Mar 2, 10.1111/bju.13465 (2016). 26935594

[b34] Vargas H. A. . Updated prostate imaging reporting and data system (PIRADS v2) recommendations for the detection of clinically significant prostate cancer using multiparametric MRI: critical evaluation using whole-mount pathology as standard of reference. Eur Radiol. 26, 1606–1612 (2016). 26396111 10.1007/s00330-015-4015-6 PMC4803633

