# Supplementary material for: The Prostate Health Index adds predictive value to multi-parametric MRI in detecting significant prostate cancers in a repeat biopsy population
Source: Sci Rep. 2016 Oct 17;6:35364. doi: 10.1038/srep35364 (PMC5066204; doi:10.1038/srep35364)
Supplement: Supplementary Information [file srep35364-s1.pdf]

The Prostate Health Index adds predictive value to multi-parametric MRI in detecting significant prostate cancers in a repeat biopsy population.

VJ Gnanapragasam[1,6,7*], K Burling[2], A George[1], S Stearn[1], A Warren[3], T Barrett[4,5], B Koo[4], FA Gallagher[4,5], A Doble[6,7], C Kastner[6,7] and RA Parker[8]

[1]Academic Urology Group, University of Cambridge, [2]Core Biochemical Assay Laboratory, Cambridge University Hospitals Trust, [3]Department of Pathology, Cambridge University Hospitals Trust, [4]Department of Radiology, University of Cambridge, [5]Department of Radiology, Cambridge University Hospitals Trust, [6]CamPARI Clinic, Cambridge University Hospitals Trust, [7]Department of Urology, Cambridge University Hospitals Trust, [8]Edinburgh Clinical Trials Unit University of Edinburgh

Supplementary Tables

Decision to biopsy	Sensitivity (95% CI)	Specificity (95% CI)	PPV (95% CI)	NPV (95% CI)	No. of negative biopsies	No. of missed cancers
mpMRI positive	0.72 (0.65 - 0.78)	0.44 (0.34 - 0.55)	0.71 (0.64 - 0.78)	0.45 (0.34 - 0.55)	53	52
mpMRI positive or PHI ≥25	0.98 (0.95 – 0.99)	0.05 (0.02 - 0.12)	0.67 (0.61 - 0.72)	0.56 (0.21 - 0.86)	90	4
mpMRI positive or PHI ≥30	0.95 (0.91 - 0.98)	0.13 (0.067 - 0.21)	0.68 (0.62 - 0.73)	0.57 (0.34 - 0.78)	83	9
mpMRI positive or PHI ≥35	0.93 (0.89 - 0.97)	0.21 (0.13 - 0.31)	0.70 (0.63 - 0.75)	0.62 (0.44 - 0.79)	75	12
mpMRI positive or PHI ≥40	0.87 (0.81 - 0.91)	0.29 (0.21 - 0.40)	0.70 (0.64 - 0.76)	0.54 (0.39 - 0.68)	67	24

Supplementary Table 1 - Diagnostic test statistics modeling each of the different strategies incorporating various PHI thresholds and detection rates for any cancer (n=279). mpMRI positive refers to lesions of Likert 3 or greater.

	Any cancer (95% CI)	Significant cancer (95% CI)
**PHI score**	0.66 (0.55 – 0.77)	0.76 (0.64 – 0.87)
**PSA score**	0.50 (0.39 – 0.62)	0.63 (0.50 – 0.77)
**PSA density**	0.42 (0.29 – 0.55)	0.62 (0.46 – 0.78)

Supplementary Table 2 –Comparative AUC of different determinants for predicting a positive biopsy for any cancer and for only significant cancers (Gleason sum ≥ 7) in men with a negative mpMRI (n=94). PSA score is based on the presenting PSA and density was derived by dividing the PSA by the mpMRI generated prostate volume.
